# College and Hospital Notes

**Published:** 1899-10

**Authors:** 


### COLLEGE AND HOSPITAL NOTES.

### OPENING OF THE UNIVERSITY OF BUFFALO.

The Medical Department of the University of Buffalo was opened for the session of 1899-1900 on Monday evening, September 25th, with the usual ceremonies. A noticeable feature of the gathering in Alumni Hall was the wearing of cap and gown by the Faculty and staff of instructors. The opening address was delivered by Dr. William H. Heath, whose remarks were especially appropriate, being directed to the students. Dr. Heath's address was largely epigrammatical and was exceptionally interesting. Some of the extracts from the address, which will be published in full in the next issue of the JOURNAL are as follows :

"Abernethy, the great surgeon, said, in his first lecture before an unusually large class : "Good God, what is to become of you?" It is not a question of what will become of you, but what may not become of you."

"Medicine is today in a more transitional state than ever before. New light is being thrown upon old phenomena; new ones discovered, and from the most unexpected quarters. Empiricism is disappearing and the art is fully established upon a scientific basis."

"Much of the just criticism upon our profession in the past which was in no small measure due to ourselves, has been corrected, by raising the preliminary educational requirements."

"It was once said that every French soldier carried a field marshal's baton in his knapsack. You may not be field marshals, but the same dispositions may lead you to fame and fortune."

"No one should attempt to enter upon the study of medicine without a suitable preliminary education. As well attempt to build a palace of stone on a foundation of mud and straw."

"Public and patient expect the doctor to be more than a medicine man; more than a detective to detect pathological changes in him; more than an accountant to strike a balance, to diagnosticate between his statement and his condition."

"No one should commence the study of medicine who is light-headed, flippant or superficial. It is a dignified calling demanding dignity. It is serious because it brings you into the heart of a family's suffering and into the shadow of death; more than the priesthood, more than any other profession."

"A level head and a judicious mind are necessary. While advantageous in all things, they are particularly so in medicine, so often the stalking ground of fads and isms, and because a scratch too much of a pencil, or a cut too deep with a knife, may poison a child or bring one nearer to death's door."

"A doctor must be many-sided. He must treat people as well as their ailments."

"Cultivate the social as well as the studious side of life. To abstain from society and be a bookworm is a mistake. Bookworms are rarely good doctors."

"Some men are so engrossed with work that they cannot take a holiday or even enjoy one. Instead of spending their declining years in well-earned leisure they break up and die for want of something to do. Some one once said that man spends one-half of his life learning how to make himself miserable the other half."

"A specialist should know everything known about something; and something about everything else. Too often he knows something about something and nothing about anything else."

"Cavities have a natural charm for the specialist and gradually his view of life and disease is limited and grows as narrow as the orifice to the particularcavity."

"Remember, that the question for you to decide is not what sort of a career is medicine, but what sort of a man you are for medicine"

"One of a physician's first duties is to collect his fees. The sick man pays best while the tears are yet wet in his eyes."

"There are two kinds of success in medicine, the scientific and the commercial or professional. It does not come to us all through lack of taste or means to become scientists. Those who endeavor to achieve professional success must look beyond the financial part. Medical fortunes are rare. Wilson probably left the largest; he left over three millions of dollars. Gull left three hundred thousand. They left the two largest fortunes in England. In this country few medical men have left large fortunes as the result of medical practice, and those who have, have combined commercial success or business sagacity with their other qualities. Many physicians with large incomes and small business tact die poor, where others with small incomes and large business tact have by successful collection and judicious investment left their family in comparative affluence."

"The abuse of medical charities is a feature you will have to contend with. The managers of these institutions in conformity with the spirit of the age, wish to make records, and benevolence is no exception. Their way of expressing this feature is by the showing as to the numbers which they treat. This is a mistake The best exemplification of progress and advance should be formed, not by its patronage, but by the character of its staff, the quality of the work done there, and especially the carefulness with which it investigates its patrons as to their ability."

"The department habit of mind is always narrowing and tends to foster sometimes ridiculous extremes and impair the power of forming by conception. As an illustration, *The Twentieth Century Practice of Medicine* has just been published and is written by as many doctors as there are subjects. The nose, for instance, is handed to an eminent authority in London, but lest the weight of it should be detracting, the nasal pharynx they sent to France, while the larynx is brought over to North America, leaving the trachea and bronchi behind, which, being considered common place, have been sent to Scotland, while the remainder of the respiratory system of the lungs has been sent to South America, handicapped, however, that it should not be considered in relation to contagious diseases; and the accessory sinuses and the bacteriological features are further distributed, one to San Francisco and the other to a Japanese student sojourning in Russia."

"The medical curriculum will look to you very formidable and almost discouraging. If discouraged think of the dial and the pendulum who were discussing matters in general, when the pendulum stopped swinging, appalled at the work before him for a year. 'It is not the stroke that I complain of,' said the pendulum, I could manage it without much fatigue, but think of the millions that I will have to do in the year.' 'Very good,' replied the face. 'But recollect that though you are thinking of a million, you are required to perform but one at a time. And that a moment will be given you to swing in.' No matter how large the curriculum, you will take it piece-meal."

"Everything comes, it is said, to him who waits. This should be corrected to 'him who knows how to wait, or hustles while he waits.' Waiting is a part of destiny. Generals do not go into battle until the opportune moment. Why should not you wait, with the world before you? Waiting is not idleness and need not be wasted. There is a time when you can exercise your duty and keep your mind and body in a state of preparation for events that follow."

The Medical Department will be largely attended this year.

---

DR. J. ACKERMAN COLES, of Newark, N. J., has made the following tender to the trustees of Jefferson College in honor of the memory of his father the late Abraham Coles, A.M., M.D., Ph. D., LL.D. (Jefferson, 1835): A Carrera marble portrait bust of William Harvey, the discoverer of the circulation of the blood. This bust and its pedestal were executed at Rome, Italy, in 1869, by the distinguished sculptor, Horatio Stone. These, with "The works of William Harvey, M. D., Physician to the King, Professor of Anatomy and Surgery to the College of Physicians, translated from the Latin, with a Life of the Author, by Robert Willis, M. D., printed for the Sydenham Society, London, 1847, I, as executor of the estate of my father, herewith proffer as gifts to Jefferson Medical College in recognition of the love and high regard he always entertained for his alma mater, its board of trustees, faculty and alumni. To these gifts I have added at the courteous suggestion of Professor J. W. Holland, M. D., a bronze medallion life-size portrait of Abraham Coles, the excellent work of the well-known sculptor, C. Conrads, of Hartford, Conn., and as further evidence of my interest in Jefferson an imported life-size bronze copy of the celebrated Roman antique statue of the boy extracting a thorn from the sole of his foot, justly admired by all critics for its beauty and anatomical accuracy."

---

A NEW training school for nervous and backward children, the first of its kind in the world, will soon be established in Chicago. It will be called the Chicago Physiological School, and its purpose will be to provide a home and school for boys and girls who are unable to cope with normal children, owing to illness or infirmity. The school will open its doors on November 1st. Miss Mary R. Campbell, of Milwaukee, will have charge. Miss Campbell was formerly in charge of the girls' department of the Wisconsin (Chippewa Falls) Institution for Feeble Minded Children. She is the author of a number of treatises on psychology.

The institution will be affiliated with the University of Chicago. The Board of Trustees will number seven, among whom are Dr. W. R. Harper and Professor George H. Mead. The number of pupils admitted to the school will be limited. Only children under fifteen years of age will be taken, preference being given to the very young. Besides instruction, medical treatment will be provided. Dr. Nicholas Senn will be invited to become a trustee.

---

DR. LILLIAN C. RANDALL announces the removal of Riverside Hospital from 306 Lafayette Avenue, to the new building, corner of Lafayette and Barton Streets, Buffalo. The structure now occupied is well adapted to the needs of the hospital, and the location is desirable. The continued prosperity of the hospital seems assured.

---

ST. VINCENT'S HOSPITAL, New York, is the first hospital to adopt the automobile ambulance. It is announced that the hospital is about to receive a gift of one of these ambulances, propelled by electricity.

